# Engineered ribosomal RNA operon copy-number variants of *E. coli* reveal the evolutionary trade-offs shaping rRNA operon number

**DOI:** 10.1093/nar/gkv040

**Published:** 2015-01-23

**Authors:** Zsuzsanna Gyorfy, Gabor Draskovits, Viktor Vernyik, Frederick F. Blattner, Tamas Gaal, Gyorgy Posfai

**Affiliations:** 1 Institute of Biochemistry, Synthetic and Systems Biology Unit, Biological Research Centre of the Hungarian Academy of Sciences, Szeged 6726, Hungary; 2 Scarab Genomics LLC, Madison, WI 53713, USA; 3 Dept. of Bacteriology, Univ. of Wisconsin-Madison, Madison, WI 53706, USA

## Abstract

Ribosomal RNA (rrn) operons, characteristically present in several copies in bacterial genomes (7 in *E. coli*), play a central role in cellular physiology. We investigated the factors determining the optimal number of rrn operons in *E. coli* by constructing isogenic variants with 5–10 operons. We found that the total RNA and protein content, as well as the size of the cells reflected the number of rrn operons. While growth parameters showed only minor differences, competition experiments revealed a clear pattern: 7–8 copies were optimal under conditions of fluctuating, occasionally rich nutrient influx and lower numbers were favored in stable, nutrient-limited environments. We found that the advantages of quick adjustment to nutrient availability, rapid growth and economic regulation of ribosome number all contribute to the selection of the optimal rrn operon number. Our results suggest that the wt rrn operon number of *E. coli* reflects the natural, ‘feast and famine’ life-style of the bacterium, however, different copy numbers might be beneficial under different environmental conditions. Understanding the impact of the copy number of rrn operons on the fitness of the cell is an important step towards the creation of functional and robust genomes, the ultimate goal of synthetic biology.

## INTRODUCTION

The copy number of the ribosomal RNA (*rrn*) operons is a characteristic trait of bacterial genomes. It influences rRNA availability, which in turn, determines the number of ribosomes, which have key roles in cellular physiology and economy. Copy numbers of *rrn* operons were found in the range of 1 to 15 in various bacterial genomes (1), and higher copy numbers tended to occur in fast-growing organisms, as well as in those with relatively large genomes (2). Large genomes have the capacity to encode multiple pathways for uptake and utilization of nutrients, promoting a life-style that readily adjusts to diverse environmental conditions. In contrast, bacteria living in stable, low-nutrient aquatic environments tended to display only a few copies of *rrn* operons, and have smaller genomes (3,4). It is thought that the *rrn* operon copy number reflects the organism's ecological strategy. However, experimental data are inconsistent, and do not resolve whether the benefit of fast growth, the capability of quick adjustment to favorable conditions, or the economic utilization of nutrients promote selection of the optimal *rrn* operon copy number.

Bacteria have evolved to maximize their growth while minimizing their adjustment to the changing conditions (5). Quick adjustment to increased nutrient supply requires the ready availability of the protein synthesis machinery, the ribosomes. Ribosome availability primarily depends on the amount of ribosomal RNA (rRNA), which regulates the expression of ribosomal proteins by a translational feed-back mechanism (6). In fast-growing *Escherichia coli* cells, up to 85% of the total RNA can be rRNA (7). To provide this rRNA supply, bacterial genomes typically include several copies of *rrn* operons in their genome, and at high growth rates most of the RNA polymerase molecules are assigned to the transcription of these operons (8).

Ribosome synthesis is energetically costly, and excess ribosome supply might result in suboptimal growth (9,10). Therefore, expression of the *rrn* operons is tightly regulated, and is quickly adjusted to the actual needs by feedback regulation mechanisms. For example, shortages of available amino acids (stringent control) and/or low initiating nucleoside triphosphate (NTP) concentrations downregulate *rrn* operon expression (11).

The genome architecture adds additional layers of complexity to the regulation of *rrn* operon expression. First, *rrn* operons are usually located near the chromosomal replication origin, and thus replicated early. Due to the multiple replication forks progressing simultaneously in fast-growing cells, their actual number can be 4–5 times higher than the nominal number in the genome sequence (7). Second, *rrn* operons are orientated in the direction of replication to avoid collision of the transcription and replication machineries (12,13). Inversion of *rrn* operons results in severe growth defects (14). Third, microscopic detection of fluorescently labeled RNA polymerase showed that *rrn* operons seem to physically condense into two foci in fast-growing cells, but take up a relaxed structure in slow-growing cells (15,16). Thus, co-ordinated long-range, dynamic changes of the genome architecture might result in a facilitated usage of the RNA polymerase pool.

Although much is known about the regulation of *rrn* operon transcription and ribosome synthesis, there are still open questions regarding the *rrn* operon number. What is the measurable contribution of *rrn* copy number to these highly regulated processes? More generally, how is the cost of ribosome synthesis balanced with the need for quick adjustment to nutrient-rich conditions and for allowing fast growth? In the era of synthetic biology, is it possible to design improved, customized (e.g., faster-growing) cells by manipulating the *rrn* operon copy number?

**Figure 1.**
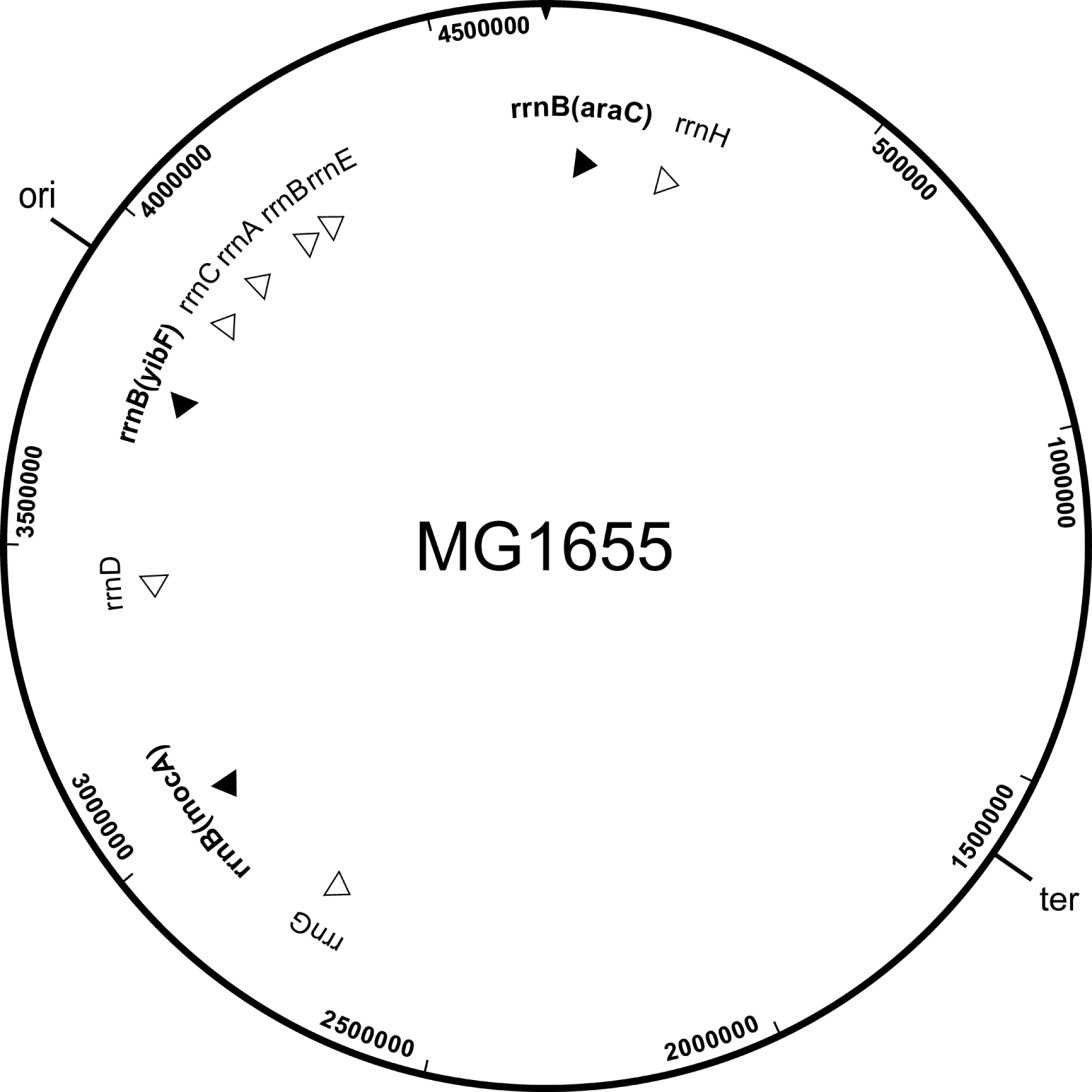
Genomic position and transcriptional direction of natural (empty arrowheads) and newly inserted (black arrowheads) *rrn* operons.

Experimental data addressing the significance of *rrn* operon copy number have been obtained mostly from studies of *E. coli*. In wild-type *E. coli* seven (1) almost identical *rrn* operons (named *rrnA* to *rrnH*) occupy conserved genomic positions (17–19) (Figure 1). To our knowledge, no genome engineering attempt has been made to increase the *rrn* operon copy number in the chromosome. When higher *rrn* copy numbers were tested, the extra operons were supplied on multicopy plasmids (9,20–22), drastically elevating the copy number, and altering the spatial distribution of the operons. For this work we avoided the problems of multicopy plasmids by creating clean insertions of extra operons directly into the genome. When *E. coli* cells were constructed with reduced numbers of *rrn* operons, it was reported that *E. coli* could grow at near maximal rate with 5 or 6 copies, and remained viable even with a single operon (23,24). Deletion of a single operon, however, resulted in longer lag time in nutritional upshift experiments, and it was concluded that all 7 operons were needed for quick physiological adaptation to favorable conditions (25). Stevenson and Schmidt determined the fitness effects of deletion of one or two *rrn* operons in competition experiments. Wild-type cells outgrew cells with genomes deleted either for *rrnB* or for *rrnA* when grown by cycles of growth in which stationary phase cells were serially re-inoculated into fresh rich medium (fluctuating conditions). It was concluded that the *rrn* operon copy number was determined by the life-history of *E. coli*, and represented an ecological strategy for adjusting to ‘feast and famine’ conditions. Surprisingly, even when the cells were grown in chemostats under nutrient-limiting, stable conditions, wt cells outcompeted the 6-operon constructs (10).

Manipulating the DNA of large, complex and secondary structure-prone *rrn* operon sequences present in multiple copies in the genome is technically challenging, and most experiments mentioned above suffer from some drawbacks (e.g., *rrn* operons were only partially removed, the tRNA pool was drastically changed as a side-effect of the deletions) (23,25). In this study, we constructed isogenic strains with 5–10 ‘clean’ (totally removed, or inserted in their totality) copies of *rrn* operons in the chromosome. Extra (new) copies of *rrn* operons were inserted in various alternative genomic sites. The effects of *rrn* operon copy number change on RNA and protein content, as well as on growth parameters were measured. To detect subtle differences in growth, cells with various *rrn* operon copy numbers were competed under two kinds of conditions thought to occur in the natural environments of *E. coli*: fluctuating, periodical influx of rich nutrients (repeated re-inoculation of batch cultures), and stable, limited influx of nutrients (glucose-limited chemostat cultures). We found evidence that the optimal copy number of the *rrn* operons is determined by the nutritional conditions and the economic utilization of the resources. Thus, while the *rrn* operon copy number of wt *E. coli* is optimized to life in a mostly fluctuating natural environment, *rrn* operon numbers other than 7 might be optimal for *E. coli* under artifically controlled nutritional conditions.

## MATERIALS AND METHODS

### Strains, plasmids and media

The prototype *E. coli* K-12 strain MG1655 (26) (NC_000913) and its multideletional derivative MDS42 (27) (NC_20518.1) were used for all cloning and recombineering experiments; intermediate constructs were created in MDS42 and operons deleted or inserted in MG1655. Plasmid constructs based on pST76-A, pSG76-C and pSG76-CS (28,29) have been applied for some genomic insertion-excision procedures, and also served as polymerase chain reaction (PCR) templates. In all experiments involving bacterial culture growth, unless indicated otherwise, standard LB or M9-glucose (0.4 m/m%) media were used. Antibiotics were used in the following final concentrations: 100 μg/ml ampicillin (Ap), 24 μg/ml chloramphenicol (Cam), 25 μg/ml kanamycin (Kan).

### Construction of strains with altered *rrn* operon numbers

Deletion of the *rrnD* operon (genomic coordinates of the deletion: 4166310–4172043) was done by our modified version of the λ Red-mediated recombineering method, described earlier (29). Briefly, a PCR-generated linear DNA fragment carrying a selectable Cam^R^ marker, I-SceI sites and terminal ‘homology boxes’ was electroporated in the cell, where it replaced the *rrnD* operon via double crossover involving the terminal ‘homology boxes’. The helper plasmid pKD46 provided the arabinose-inducible recombinase functions. The chromosome of the selected recombinant cells was then cleaved by I-SceI at its 18-bp recognition sites present on the integrated fragment, by expression induced from helper plasmid pSTKST. RecA-mediated intramolecular recombination then repaired the break and removed exogenous sequences via cross-over between an internal ‘homology box’ and a flanking chromosomal segment. Generation of the desired, scarless deletion was confirmed by PCR using flanking primers. Deletion of *rrnB* (genomic coordinates of the deletion: 3423257–3429197) was performed in a similar way, except that the final step, removal of the exogenous sequences, was not succesful, and a Cam^R^ marker was left in the genome.

Construction of extra *rrn* operons was a multistep procedure. To engineer an intermediate *rrn* insertion construct, long flanking segments were generated at the *rrnB* donor and at the *yibF* acceptor sites to serve as ‘homology segments’ for λ Red-mediated recombineering in MDS42 (Supplementary Figure S1). First, a ∼1-kb PCR-generated segment, representing the downstream region of the *yibF* target site (GH, genomic coordinates 3759619–3758571) was fused to a segment representing the downstream region of *rrnB* (EF, genomic coordinates 4169561–4170300), cloned in pST76-A, and the plasmid was inserted downstream of *rrnB* by a single cross-over. Next, a ∼1-kb fragment representing an upstream sequence of the *rrnB* operon (AB, genomic coordinates 4163047–4163783) was fused to a fragment representing the upstream region of the target site (CD, genomic coordinates 3768945–3767872), cloned in pSG76-C, and inserted in the acceptor site by recombination via the CD fragment. An AB-GH segment, encompassing the *rrnB* region (from 899 basepairs upstream of the *rrnB* P1 transcription start site to 171 basepairs downstream of the T2 terminator U-track) was then generated from the donor site by PCR, and recombined by λ Red (expressed from pKD46) via the 1-kb terminal homologies (AB and GH) into the acceptor site. Finally, to create and intermediate construct for all subsequent steps, the EF to GH segment encompassing the inserted pST76-A plasmid was replaced at the acceptor site by recombineering, using a PCR-generated fragment composed of EF’-GH’ (genomic coordinates 4169561–4169606 and 3759619–3759573, respectively), pSG76-CS (carrying I-SceI sites and a Cam^R^ marker) and GH.

**Figure 2.**
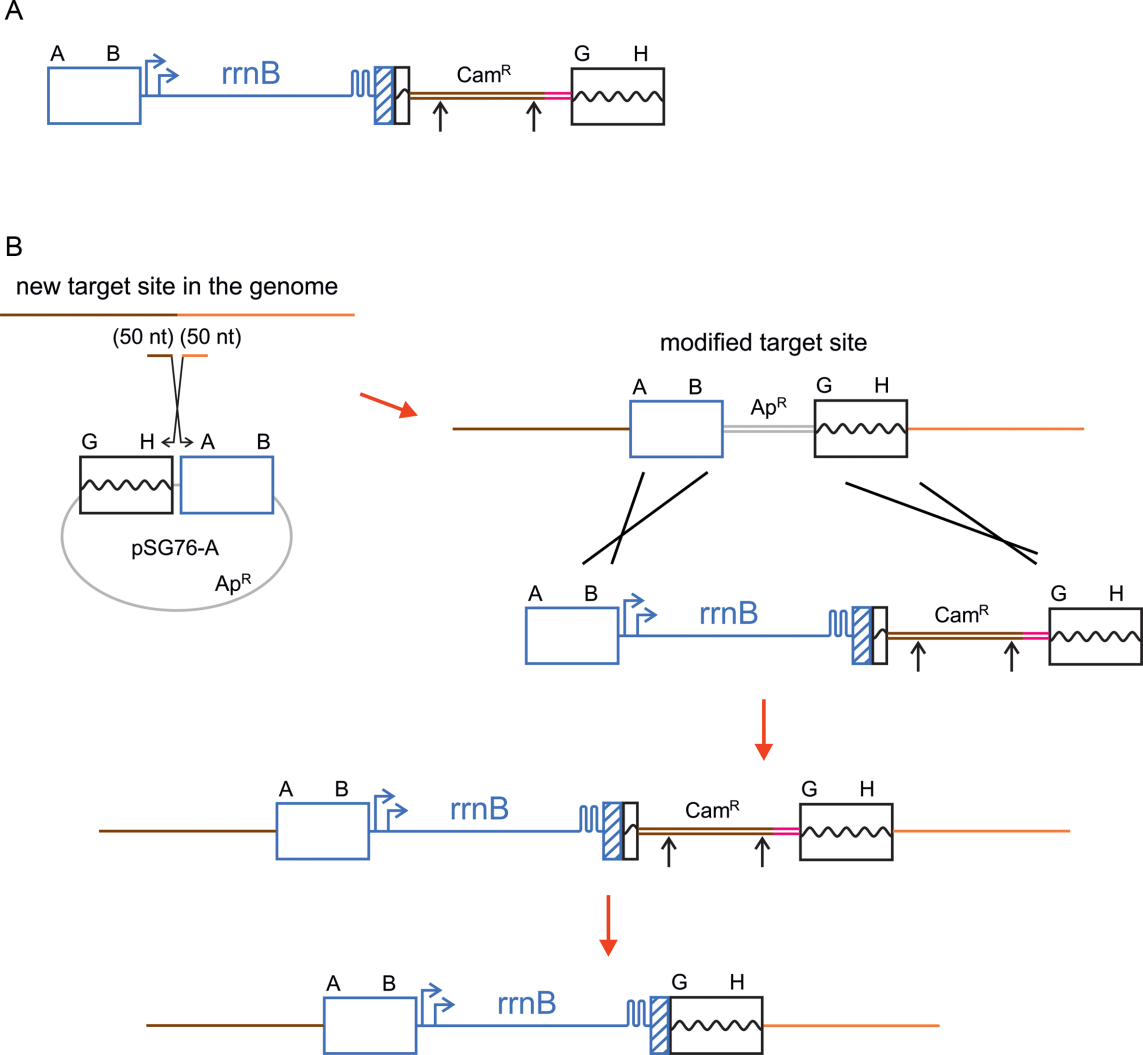
Construction of MG1655 strains with extra *rrnB* operons. (**A**) The intermediate construct harboring a new copy of *rrnB* with a downstream marker gene (for the construction steps see Supplementary Figure S1). (**B**) Preparation of a landing pad at an arbitrary genomic site of MG1655, insertion of the intermediate *rrnB* construct into the landing pad, and scarless removal of the antibiotic resistance marker gene. Blue color indicates genomic sequence copied from the *rrnB* region (blue arrows represent *rrn* promoters, and hairpins represent T1, T2 terminators). Red arrows indicate λ Red-mediated recombineering steps. Boxes indicate homology arms participating in the recombination events (genomic coordinates in Materials and Methods). Black arrows represent I-SceI sites.

This intermediate construct (Figure 2a) gave rise to a clean *rrnB* insertion at this site by scarless removal of exogenous plasmid sequences via I-SceI (expressed from helper plasmid pSTKST) cleavage, followed by intramolecular recombination between GH and GH’. The construct was also used as a starting point for all other *rrn* operon insertions (Figure 2b), proceeding in four steps: (i) preparation of a marked AB - GH ‘landing pad’ at the new acceptor site by λ Red recombineering of a linear DNA fragment (generated from a pSG76-A plasmid carrying AB-GH using PCR primers with 50-bp 5′ homologies to the target site) into the genome, (ii) generation of an AB-*rrnB*-pSG76CS-GH PCR fragment from the intermediate construct above, (iii) recombineering the AB-*rrnB*-pSG76CS-GH fragment into the acceptor site via the AB and GH homologies, (iv) scarless removal of exogenous plasmid sequences via I-SceI cleavage, followed by intramolecular recombination.

### Measurement of RNA and protein content

RNA and protein content was essentially measured as described (30,31). Briefly, at OD_600_ = 0.4–0.5, cells (15 ml) were chilled on ice. Aliquots were used for isolation of genomic DNA using E.Z.N.A. Bacterial DNA Isolation Kit. The rest of the cells were collected by centrifugation (10 min, 4000 rpm), washed and resuspended in 15 mM Tris HCl/4 mM EDTA/4 mM dithiothreitol. Cell extracts were prepared by sonication. Protein content of an aliquot was determined by an improved Bradford assay (32). From the rest of the cell extract, nucleic acids and protein was precipitated by 10% trichloroacetic acid (TCA) on ice. RNA was then dissolved in 5% TCA at 90°C. The RNA concentration was measured in a spectrophotometer using the solvent as blank.

### Measurement and calculation of growth parameters

To measure growth parameters, a Synergy HT automated microplate reader machine (BioTek, USA) was used. 1 μl inocula from overnight starter cultures were transferred into 100 μl fresh medium in dedicated 96-well plates. For each construct, OD_600_ was measured every 5 min for 24 h at 37°C with continuous shaking. Growth parameters (doubling time, lag phase time) were calculated by using previously described methods (33).

### Competition of strains in batch and chemostat cultures

For batch culture competition experiments, overnight starter cultures of the wt and the competing strain were mixed at 1:1 volume, then 400 μl of the mix was inoculated into 100 ml LB or M9-minimal medium, and grown in a 500-ml Erlenmeyer flask with shaking at 225 rpm at 37°C. 400 μl of the culture was then daily (up to 5 days) transferred into fresh medium.

For chemostat competition experiments, overnight starter cultures of the wt and the competing strain were grown separately in M9 minimal medium supplemented with 0.2 m/m% glucose and 0.03 m/m% PEG6000. The two cultures were then mixed at 1:1 ratio, and 1 ml of the mix was inoculated into 600 ml of the same medium, and grown in the chemostat as a batch culture for 15–17 h (∼10 generations) with 1.1 l/min aeration, at 500 rpm (mixing rotor), at 37°C. At this stage (0 time point), the chemostat function was started, and cells were grown for an additional 5 days (up to 100 generations) at a dilution rate of 0.5 per h, using the same medium.

To track the progress of competitions, unless indicated otherwise, samples were taken at every re-inoculation of batch cultures or at every 24 h from chemostat cultures. A *lacZ^−^* variant of MG1655, where the LacZ activity was inactivated by a targeted incorporation of a nonsense mutation (34) was used for differentiating the competing strains. Cell counts of each competitor were determined by spreading appropriate dilutions on MacConkey agar plates.

Fitness differences were estimated by calculating the selection coefficients of the altered *rrn* copy number strains. The natural logarithm of the ratio of the modified and the parental strain was plotted against the number of generations, where the slope of the linear regression line is the selection coefficient of a given strain (35).

## RESULTS

### Construction of isogenic strains with altered *rrn* copy numbers

A list of MG1655 derivatives with altered *rrn* operon copy numbers (5–10) are shown in Table 1.

**Table 1.** Strains with various *rrn* copy numbers

Name	rrn status^a^
rrn5(ΔBD)	*rrnB* and *rrnD* deleted
rrn6(ΔB)	*rrnB* deleted
rrn6(ΔD)	*rrnD* deleted
rrn7	wild-type
rrn8(araC)	*rrnB* copy inserted at *araC* (71267)
rrn8(yibF)	*rrnB* copy inserted at *yibF* (3761992)
rrn8(mocA)	*rrnB* copy inserted at *mocA* (3015910)
rrn9(araCyibF)	*rrnB* copies inserted at *araC* and *yibF*
rrn10(araCyibFmocA)	*rrnB* copies inserted at *araC, yibF* and *mocA*

^a^New copies of rrnB were inserted downstream of the coordinates shown in parentheses.

Operons *rrnB* and *rrnD* were deleted both separately and in combination, resulting in strains rrn6(ΔB), rrn6(ΔD) and rrn5(ΔBD), respectively. Both operons carry redundant tRNA genes that were also present elsewhere in the genome. In the case of the *rrnB* deletion, the attempt to remove the exogenous sequences failed despite several tries, and a Cam^R^ gene was left in the genome between *murI* and *murB*.

To increase the *rrn* operon number, an extra copy of *rrnB*, together with marker sequences, was integrated into the genome (Figure 2a). This construct, called the insertion intermediate, was then used as a template for all further *rrnB* insertions into parental MG1655 (Figure 2b). The procedure involved the insertion of a ‘landing pad’ (a marked, genomic segment pair) into virtually any preselected intergenic site, then recombination of the *rrnB* copy into this landing pad, followed by removal of the exogenous marker sequences. The extra *rrnB* copies were designed to be co-oriented with the natural rrn operons, and placed into the ori-proximal half of the genome. Three alternative 8-operon variants, rrn8(mocA), rrn8(yibF) and rrn8(araC) (named after the genes neighboring the *rrn* insertion site), as well as a 9- and a 10-operon combinations were constructed in MG1655. We note that attempts to insert a copy of *rrnB* in orientation opposite to replication failed (i.e. cells with an inverted operon were non-viable) at both the *rrnD* and *araC*. This adds further support to previous studies showing that head-on collisions of the transcriptional and translational apparatus (caused by rRNA operon inversions) are severely detrimental to cell survival (36,37). A combined genomic map of the natural and artificially manipulated *rrn* operons is shown in Figure 1.

### RNA and protein content of cells reflect rrn operon copy numbers

**Figure 3.**
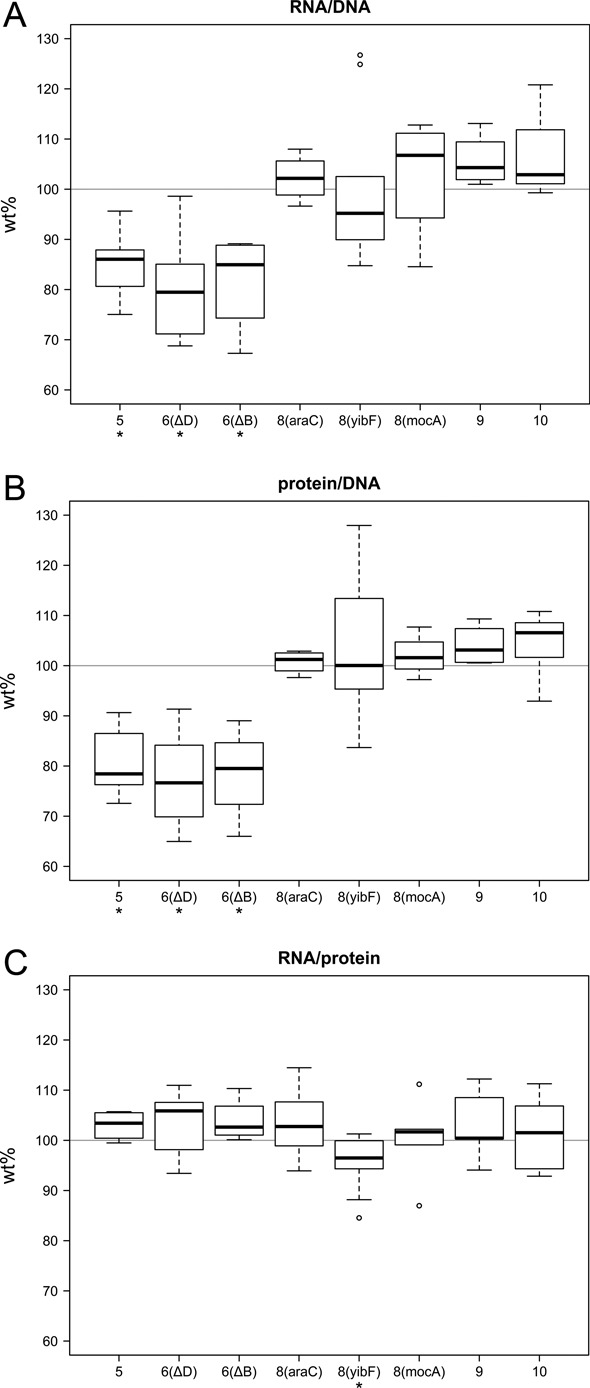
RNA and protein content of the cells with various *rrn* operon numbers, compared to the values of wt (MG1655) cells (100%). The RNA (**A**) and protein (**B**) content is normalized to DNA content. RNA:protein ratios are shown in (**C**). Strains are labeled by their *rrn* operon number and the site of the operon modification. Center lines show the medians; box limits indicate the 25th and 75th percentiles; whiskers extend 1.5 times the interquartile range; and outliers are represented by circles. At least five measurements per strain were performed (compared to the wt in each experiment). Asterisks indicate significant difference compared to the wt (*P < 0.05, paired samples *t*-test).

Earlier studies indicated that drastic increases in the rRNA gene dosage might be partially compensated by transcriptional downregulation of the stable RNA promoters (9,20,38). To test whether moderate changes in *rrn* copy number would tip the balance of regulation of rRNA and ribosome availability, *rrn* operon copy number variants were sampled at mid log growth phase (OD_600_ = 0.4–0.5) and their total RNA and protein content analyzed. Data were normalized for DNA content (Figure 3a and b), paralleling thus per cell values. Both RNA and protein content of 5- and 6-operon cells were significantly lower than that of the wt. Values obtained with 8–10 operons were generally higher, but not differing significantly from those of the wt. Regardless of *rrn* operon number, the RNA:protein ratio was essentially similar in the strains (Figure 3c).

### Cell size increases with more *rrn* operon copies

**Figure 4.**
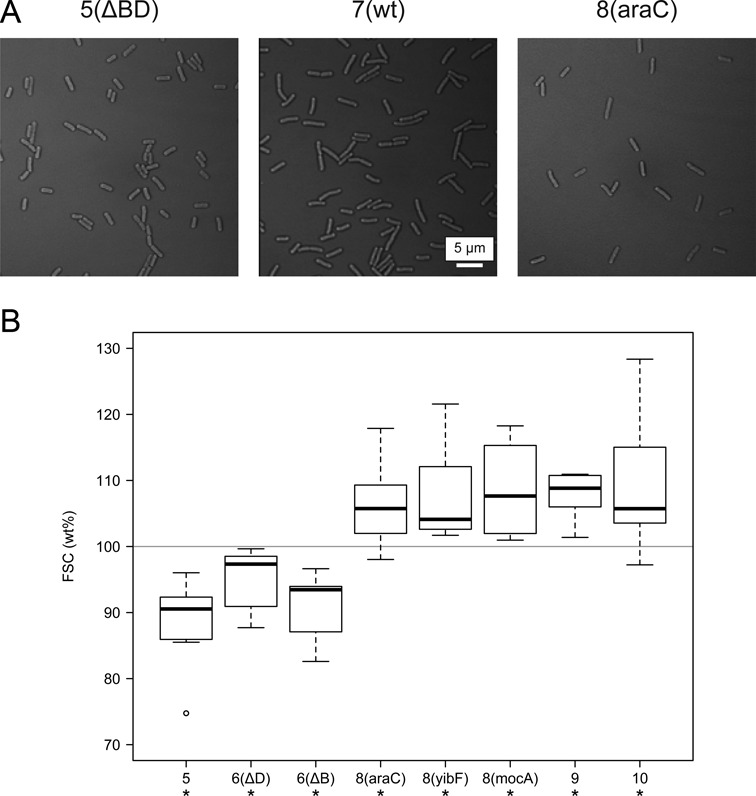
(**A**) Microscopic images of selected strains. (**B**) The size (forward scatter, FSC) of the modified cells was measured by flow cytometry and compared to the wt in each measurement. Center lines show the medians of relative size; box limits indicate the 25th and 75th percentiles; whiskers extend 1.5 times the interquartile range; and outliers are represented by circles. Results are based on seven independent experiments (20 000 cells per experiment). Asterisks denote significant difference compared to the wt (*P < 0.05, paired samples *t*-test).

Cell morphology of selected constructs was studied both by light microscopy (Figure 4a) and flow cytometry. Aliquots of cells were taken for analysis at identical growth phase (LB, 37°C, OD_600_ = 0.4–0.5). According to microscopic measurements, relative cell dimensions (length/width) did not change significantly (data not shown). Based on the forward scattering light parameter (FSC), paralleling the *rrn* operon copy number, a general increase in cell size was observed. The 5- and 6-operon cells were significantly smaller, and 8–10-operon cells were significantly larger than wild-type cells (Figure 4b).

### Growth parameters measured in rich medium decline with deviations from the wt *rrn* operon number

**Figure 5.**
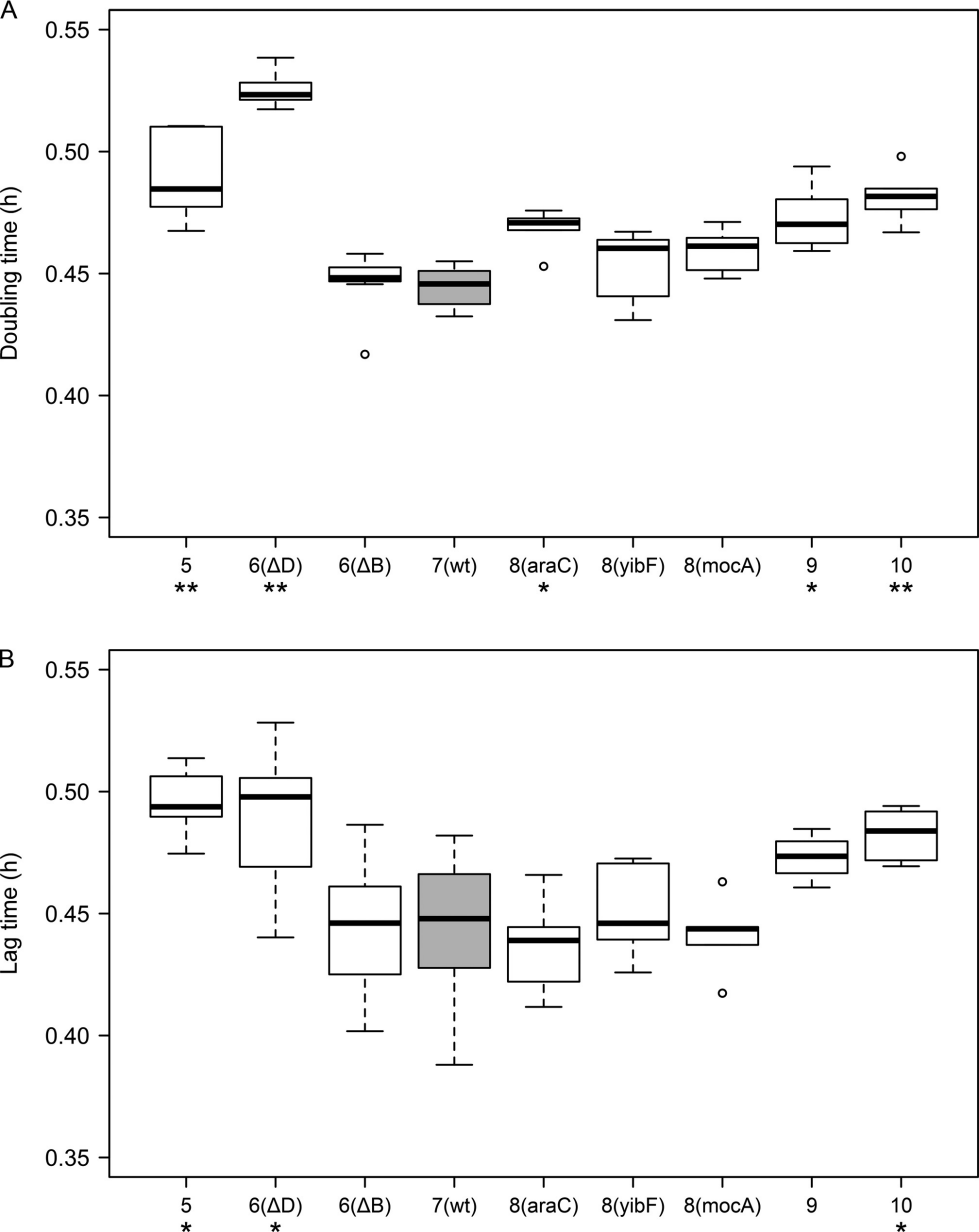
Growth parameters of the strains grown in rich (LB) medium. Doubling time (**A**) and lag time (**B**) of cells are shown (at least 5 biological replicates per strain with 40 technical replicates each). Center lines show the medians; box limits indicate the 25th and 75th percentiles; whiskers extend 1.5 times the interquartile range; and outliers are represented by circles. Asterisks show significant difference compared to the wt (*P < 0.05, **P < 0.001; one-way ANOVA with *post hoc* Dunnett's test).

Growth parameters (doubling time, and lag time for the transition from stationary phase to exponential growth) were measured in rich medium in batch cultures (Figure 5a and b). Both parameters showed the same tendency: while values of rrn6(ΔB) and the 8-operon constructs were similar or showed little change compared to that of the wt, they increased for both lower and higher operon number (5,9,10) constructs. We noted that the two 6-operon constructs differed from each other: while deletion of *rrnB* caused only minor changes, deletion of *rrnD* resulted in much more pronounced effects.

### Nutrient availability influences the competitive advantage of *rrn* operon number variants

**Figure 6.**
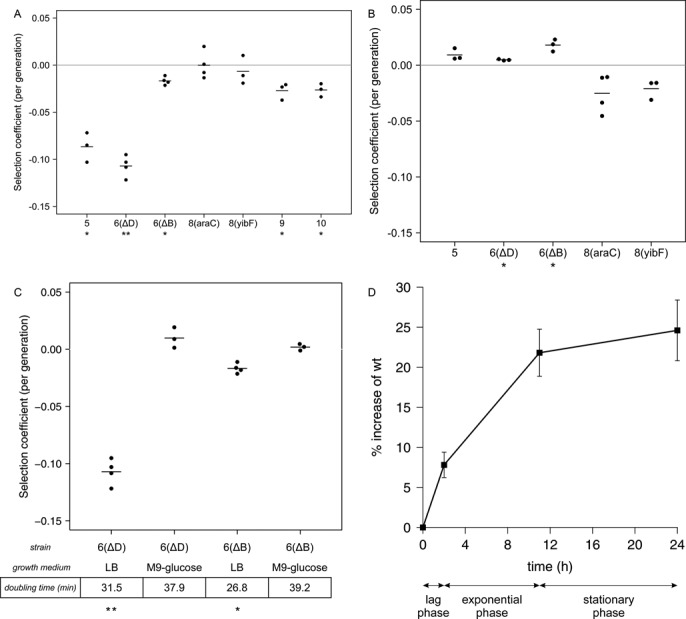
Selection coefficients of the strains with various *rrn* copy numbers during pairwise competitions with wt cells in batch cultures (**A** and **C**) and in a chemostat (**B**). The medium used in the experiments was LB (A), M9-glucose (B), or as indicated (C). Positive values mean that the winner of the competition is the modified strain. Dots represent results of individual experiments, averages are shown by lines. Asterisks mark significant difference compared to the wt (*P < 0.05, **P < 0.001; one-sample *t*-test against zero). (**D**) Progress of competition between the wt strain and rrn6(ΔD) in batch cultures in rich medium (LB), resolved at different growth phases. The % change of wt in a single growth cycle is shown. Samples were taken immediately after re-inoculation (0 time point), at the end of lag phase (2 h) and at the beginning of stationary phase (11 h). Values are averages of six independent 3-day experiments; error bars show standard errors of the means.

To detect slight differences in fitness, strains with altered *rrn* operon number were competed with wt for growth in either fluctuating (serial reinoculation in LB medium in flasks) and in stable (growth in chemostat in M9 minimal salts/glucose medium) nutritional conditions. Under fluctuating conditions, selection coefficients, with respect to *rrn* operon number, revealed the following trend in competition success: 5,6<7,8>9,10 (Figure 6a). These results show that optimal response to rich nutrient influx requires a high number (up to 7–8) of *rrn* operons, but unnecessarily high numbers (9,10) might be disadvantageous if the amount of energy consumed by ribosome production is too high. Under limiting and stable nutritional conditions, a markedly different trend of competitive efficiency was observed: 5,6>7>8 (Figure 6b), that is, an operon number lower than 7 is preferred for slow growth at steady conditions. Competitive potential of the 6-operon strains rrn6(ΔB) and rrn6(ΔD) followed the same trend, but to different extents.

### Adaptation to nutrient availability and the capacity of rapid growth both contribute to the selection of the optimal *rrn* operon number

Growth parameter measurements and results of the competition experiments suggested that both the advantage of quick adjustment to nutrient upshifts, and the capacity for rapid growth might act as selection forces determining the optimal *rrn* operon number. To confirm this, selected strains (rrn6(ΔB) and rrn6(ΔD) vs. wt) were competed at two different growth rates: batch culture competition experiments in rich medium were complemented by competitions performed in minimal medium (Figure 6c). Results showed that the quality of the medium influenced the competitive advantage: at slower growth in minimal medium, the advantage of the wt strain no longer existed. In fact, the 6-operon strains slightly outcompeted the wt strain. To separate the effects of the adjustment to fresh nutrients and the capacity for rapid growth, the progress of competition between rrn6(ΔD) and wt was further analyzed at consecutive growth phases in rich medium (Figure 6d). Results clearly show that the competitive fitness difference between the 7- and the 6-operon strain varies with the growth phases: it is most pronounced during the lag phase, provides further advantage during exponential phase, and ceases at stationary phase. Together, the results suggest that under different conditions (depending on the quality of the nutrients, and the dynamics of their availability), different numbers of *rrn* operons are optimal to provide the proper number of ribosomes in a timely manner.

## DISCUSSION

Construction of *E. coli* with altered *rrn* copy number is not a trivial task. Previous studies aimed at reducing the *rrn* copy number were prone to unwanted side-effects not directly related to the *rrn* copy number: *rrn* operons were inactivated by insertions or were only partially deleted, leaving regulatory sequences in place, the tRNA pool was inadvertantly changed by the operon removal (23,25), large genomic segments were transferred between different strains jeopardizing isogenicity, or strains physiologically adapted to specific conditions were used (10). When the effects of higher than normal *rrn* copy numbers were measured, the extra operons were supplied on multicopy plasmids, drastically changing the stoichiometry (9,20,21,38).

We presented here altered *rrn* copy-number genomic constructs that were created with great precision and designed to minimize side-effects of the deletions and insertions. First, *rrnB* and *rrnD* harbor extra copies of tRNA genes (*gltT* and *ileU/alaU/thrV*, respectively) that are also present in other genomic locations, thus deletion of them, or insertion of extra copies of *rrnB* would be expected to cause only minimal change in the tRNA pool. Second, deletions or insertions encompassed the *rrn* operons in their entirety, from upstream regulatory elements and promoters to downstream terminators. By deleting either *rrnB, rrnD* or both, as well as inserting extra copies of *rrnB* in three alternative genomic locations, a set of largely isogenic strains with 5–10 *rrn* operons were created.

The *rrn* operons and their products, ribosomes, are key players in cellular physiology, and changes in their gene dosage might influence macromolecular composition of the cell. On the other hand, it has been shown that copy number changes are compensated by transcriptional regulation: deletion of an operon causes upregulation of the remaining ones, and addition of extra (multicopy plasmid-borne) operons cause downregulation of them (21,38,39). We measured the total RNA (the vast majority of it being rRNA) and protein content of the various strains, and made three observations: first, the RNA:protein ratio of the cells remained essentially constant, a consequence of the cell's homeostasis. Second, with increasing operon numbers, an increase in RNA and protein content per cell was observed. The change was significant in the 5–7 operon range, but diminished for extra operons. Third, in parallel with the RNA and protein content increase, there was an increase in the size of the cells.

Growth parameters, measured in rich medium (LB), showed subtle or moderate changes among the various strains. Doubling time and lag time showed little differences between 7- and 8-operon constructs. Both lower and higher *rrn* operon numbers (5, 6, and 9, 10) resulted in an increase of the doubling and lag phase times. These results are partially in line with earlier findings (10,25) showing that, compared with wt, 5- or 6-operon cells can grow at near maximal rate, but their lag times are longer. Unexpectedly, effects of deletion of *rrnB* and *rrnD* on growth were significantly different. While the growth parameters of rrn6(ΔB) were similar to that of the wt, rrn6(ΔD) displayed significantly longer lag time and doubling time.

For more sensitive detection of fitness differences, strains were competed with wt cells under both fluctuating (repeatedly re-inoculated batch cultures) and stable, nutritionally limited conditions (chemostat cultures). The observed competition successes revealed a clear pattern. In the fluctuating environment, 7 or 8 *rrn* operons were most efficient, and both lower (5 and 6) and higher *rrn* operon numbers (9 and 10) were disadvantageous. In stable nutrient influx conditions, in contrast, wt cells outcompeted the 8-operon strains, and wt was outcompeted by lower (5,6) rrn operon constructs. While the batch culture experiments are in agreement with other studies (10,25), the chemostat results contrast with an earlier work (10), where cells deleted for either *rrnA* or *rrnB* were outcompeted to various extent by wt cells in similar chemostat competitions. We note, however, that the parental strain used in that study had been evolved in the laboratory for 10 000 generations specifically to take advantage of periodic fluctuations in resource availability. Moreover, during construction of those deletion strains, P1 transduction between heterogenic *E. coli* strains was applied, raising the possibility of compromised isogenity.

To dissect further the factors promoting the selection of the optimal number of *rrn* operons, batch culture competitions were performed using either rich and poor media. At slow growth, differences in competitiveness decreased. Furthermore, we showed that the competitive advantage depends on the growth phases. Together, these results indicate that the optimal number of *rrn* operons is determined by the advantage of both quick adjustment to nutrient influx and the capacity for rapid growth.

The exact molecular mechanism determining the *rrn* copy number remains unknown. It seems likely that, despite the feedback regulation of rRNA transcription, 7 operons provide some excess of rRNA (and therefore ribosomes) under stable and slow growth conditions, causing an energetic handicap in competition with a 5- or 6-operon strain. In contrast, for maximal adaptation to drastic nutritional upshifts, fewer than 7–8 operons cannot supply enough rRNA. Increasing the operon number further (9,10), however, does not add to the rapid response (presumably due to the limits of the metabolism of the cell), and might even be energetically costly, causing physiological imbalance in the cell. Additionally, according to a coarse-grained model of macromolecular synthesis in *E. coli*, molecular crowding effects might set the limit on the optimal copy number of the *rrn* operons (40). These effects could explain the reduced growth seen at high (plasmid-borne) *rrn* gene dosage. It is interesting to note that the cell size seemed to parallel the change in RNA and protein content. The optimal *rrn* operon number thus might be a consequence of coupling with an optimal cell surface to volume ratio as well.

Changes in the physiological and competitive characteristics due to deletion of *rrnB* or *rrnD* followed the same trend, but to different extents. This might be due to the fact that the contribution of the promoter elements to *rrnD* expression is different from that of *rrnB* (41). An alternative explanation is that, although the tRNAs encoded by *rrnB* and *rrnD* are redundant, they might not be expressed to the same level at their other genetic locations. Another possibility is that the difference is due to the number of 5S rRNA genes deleted. While *rrnB* contains one 5S rRNA gene, *rrnD* carries two copies, and it was previously shown that the loss of 5S rRNA genes decreases cell fitness more rapidly than loss of a similar number of 16S and 23S rRNA genes (42). Effects of *rrnB* and *rrnD* deletions were not additive in the double deletion mutant. In fact, deletion of both operons caused smaller changes in doubling time and lag time, as well as in RNA and protein content, than deletion of *rrnD* alone. The cause of this is unknown, but similar positive epistatic interactions have been described in the literature (24). The three alternative 8-operon insertions, despite the fact that they were placed in different genomic locations (different macrodomains, different distances from *ori*), were functionally similar, revealing no obvious genome architectural effects.

Our results suggest that environments with relatively poor, but stable nutritional conditions would select fewer than 7 *rrn* operons over time. On the other hand, the 7- and the 8-operon versions were the most effective competitors in fluctuating, nutrient rich conditions. The results confirm experimentally that the *rrn* operon copy number (7) of wild-type *E. coli* is optimized to living conditions mostly involving feast and famine periods but also reflect an evolutionary compromise ensuring maximal growth with minimal adjustment to occasionally nutrient-poor, stable environments as well.

The data presented here might contribute to the refining of system-level models of bacterial growth (40,43). Custom-designed cells with streamlined or synthetic genomes are already being designed for specific purposes. Adjustment of this distinctive bacterial trait, the number of *rrn* operons, to the desired environmental conditions is a factor to be taken into account at the design stage of customization for the most appropriate tailoring of the new genome.

## SUPPLEMENTARY DATA

Supplementary Data are available at NAR Online.

SUPPLEMENTARY DATA
