# Supplementary material for: Engineered ribosomal RNA operon copy-number variants of E. coli reveal the evolutionary trade-offs shaping rRNA operon number
Source: Nucleic Acids Res. 2015 Jan 23;43(3):1783–94. doi: 10.1093/nar/gkv040 (PMC4330394; doi:10.1093/nar/gkv040)
Supplement: SUPPLEMENTARY DATA [file supp_43_3_1783__index.html]

Engineered ribosomal RNA operon copy-number variants of E. coli reveal the evolutionary trade-offs shaping rRNA operon number — SUPPLEMENTARY DATA 

# Engineered ribosomal RNA operon copy-number variants of *E. coli* reveal the evolutionary trade-offs shaping rRNA operon number

## SUPPLEMENTARY DATA

**Files in this Data Supplement:**

- SUPPLEMENTARY DATA
