# Supplementary material for: Engineered ribosomal RNA operon copy-number variants of E. coli reveal the evolutionary trade-offs shaping rRNA operon number
Source: Nucleic Acids Res. 2015 Jan 23;43(3):1783–94. doi: 10.1093/nar/gkv040 (PMC4330394; doi:10.1093/nar/gkv040)
Supplement: SUPPLEMENTARY DATA [file supp_gkv040_nar-03486-v-2014-File002.pdf]

Figure S1.

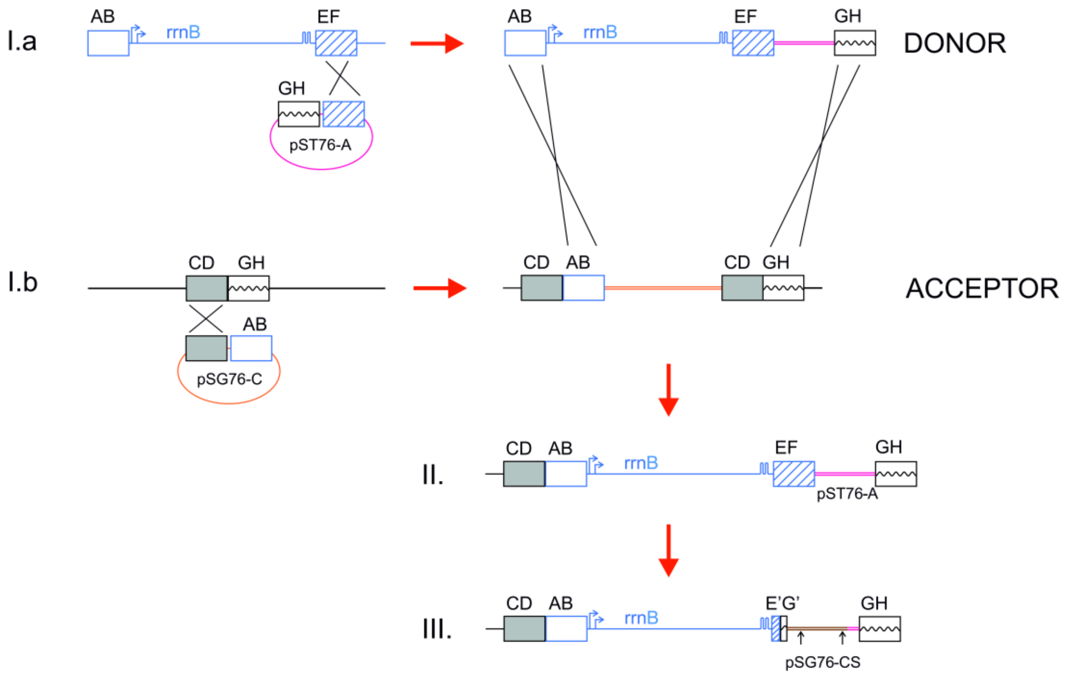


Engineering steps of the initial, intermediate construct harboring a new copy of *rrnB* with a downstream marker gene.

I.a. Insertion of GH homology box (using pST76-A plasmid) next to the *rrnB* operon/donor site.

I.b. Insertion of AB homology box (using pSG76-C plasmid) next to the acceptor/new insertion site.

II. PCR amplification of the modified *rrnB* region (AB-rrnB-pST76-A-GH), and insertion into the modified acceptor site using AB and GH homology boxes.

III. Replacing the pST76-A plasmid with a pSG76-CS-derived linear fragment containing the Cam$^R$ gene, two I-SceI sites, and a short homology region to GH (G').
